# Hyaluronic Acid Prevents Fusion of Brain Tumor-Derived Spheroids and Selectively Alters Their Gene Expression Profile

**DOI:** 10.3390/biom14040466

**Published:** 2024-04-10

**Authors:** Irina Arutyunyan, Anna Soboleva, Dorzhu Balchir, Enar Jumaniyazova, Vera Kudelkina, Andrey Elchaninov, Timur Fatkhudinov

**Affiliations:** 1 National Medical Research Center for Obstetrics, Gynecology and Perinatology Named after Academician V.I. Kulakov Ministry of Healthcare of the Russian Federation, 4 Oparina Street, 117997 Moscow, Russia; 2 Research Institute of Molecular and Cellular Medicine, RUDN University, 6 Miklukho-Maklaya Street, 117198 Moscow, Russia; 3 Avtsyn Research Institute of Human Morphology of Federal State Budgetary Scientific Institution Petrovsky National Research Centre of Surgery, 3 Tsyurupy Street, 117418 Moscow, Russia

**Keywords:** high-grade brain tumors, tissue strains, 3D culture, spheroids, hyaluronic acid, spheroid fusion

## Abstract

Hyaluronic acid (HA), a major glycosaminoglycan of the brain extracellular matrix, modulates cell behaviors through binding its receptor, Cd44. In this study, we assessed the influence of HA on high-grade brain tumors in vitro. The model comprised cell cultures derived from six rodent carcinogen-induced brain tumors, forming 3D spheroids prone to spontaneous fusion. Supplementation of the standard culture medium with 0.25% HA significantly inhibited the fusion rates, preserving the shape and size uniformity of spheroids. The 3D cultures were assigned to two groups; a *Cd44lo* group had a tenfold decreased relative expression of *Cd44* than another (*Cd44hi*) group. In addition, these two groups differed by expression levels of *Sox2* transcription factor; the correlation analysis revealed a tight negative association for *Cd44* and Sox2. Transcriptomic responses of spheroids to HA exposure also depended on *Cd44* expression levels, from subtle in Cd44lo to more pronounced and specific in Cd44hi, involving cell cycle progression, PI3K/AKT/mTOR pathway activation, and multidrug resistance genes. The potential HA-induced increase in brain tumor 3D models’ resistance to anticancer drug therapy should be taken into account when designing preclinical studies using HA scaffold-based models. The property of HA to prevent the fusion of brain-derived spheroids can be employed in CNS regenerative medicine and experimental oncology to ensure the production of uniform, controllably fusing neurospheres when creating more accurate in vitro brain models.

## 1. Introduction

Currently, several options exist for preclinical in vivo models of brain tumors. The most common protocol involves carcinogenic chemical exposure and has been in use for half a century. Experimental tumors may be induced through local, oral, intravenous, or transplacental exposure of carcinogenic compounds into newborn, adult, or pregnant rodents or other animals [1]. Years of active use of this model resulted in unique collections of transplantable tissue strains of high-grade brain tumors; one of them is stored in the Laboratory of Neuromorphology of Avtsyn Research Institute of Human Morphology of Federal State Budgetary Scientific Institution “Petrovsky National Research Centre of Surgery”, Moscow, Russia.

The formation of carcinogen-induced tumors is a random process, making it impossible to predict the type of damaged progenitor cells and the genes affected by mutations. Therefore, for convenience, almost immediately after the development of an in vivo model, 2D cell lines were isolated from induced brain tumors. This is how well-characterized rat (C6, 9L, T9, RG2, F98, BT4C, RT-2, and CNS-1) and mouse (GL261, SMA-560, CT-2A, SB28) glioma cell lines, often used as simple and accessible in vitro models in experimental neuro-oncology and preclinical studies, were obtained [2,3].

Despite the fact that 2D cell cultures remain the most common in vitro tumor model, recent years have witnessed the active development of brain 3D modeling, making it possible to obtain multicellular volumetric structures. Such models include spheroids, organoids, tissue engineering models, bioprinted models, and organotypic slices [4]. Most of the 3D options harbor or mimic the extracellular matrix (ECM) components known to modulate cell fate during development or malignization [4]. Both synthetic and natural polymers are used for in vitro modeling of normal or tumor nervous tissue. The advantages of synthetic matrices include the ability to provide specified biomechanical properties, while natural polymeric scaffolds are more relevant due to the maintenance of important biochemical cues [5]. Obviously, an ideal scaffold should combine these advantages, and the most suitable candidate for this role, hyaluronic acid (HA), the major component of the brain ECM, is primarily synthesized by neurons and glial cells [6]. HA is a linear anionic, nonsulfated glycosaminoglycan that can be subjected to chemical modification, leading to the formation of cross-links between polymer chains and a change in its mechanical properties, which makes it possible to obtain convenient hydrogel scaffolds. Moreover, some chemical modifications of the HA molecule create an opportunity to obtain water-insoluble polymers, which implement to expand the range of produced scaffolds, for example, to manufacture membranes, gauzes, nonwoven meshes, microspheres, tubes, etc. Biocompatible HA-based scaffolds and hydrogels are used extensively as wound-covering devices or soft tissue fillers and as scaffolds for the tissue engineering of normal tissue, including skin, cartilage, bone, and nerve reconstruction [7,8].

The localization and deposition of HA in brain tissue occurs around myelinated fibers and gives rise to lattice-like ECM substructures, called perineuronal nets, that surround neuronal cells and aid in developmental neuroplasticity and brain maturation [9]. Apart from its spacing and organizing roles, HA can modulate cell behaviors, such as migration, proliferation, differentiation, and inflammation, through binding cell-surface receptors [9,10]. Numerous studies confirm that HA plays an important role in different physiological and pathophysiological conditions, such as water homeostasis, angiogenesis, inflammatory processes, tumorigenesis, the evasion of apoptosis, and even multidrug resistance [8]. For example, nanoparticles decorated with HA could be a potential delivery system for anticancer drugs to target tumor cells overexpressing the CD44 transmembrane receptor, thus reducing the side effects of conventional therapies [11]. It has been shown that 3D models of brain tumor tissue, in which tumor and tumor-associated cells interact with hyaluronic acid as ECM, are more relevant for predicting response to chemotherapy [12,13]. In addition, data on the response of brain tumor cells (for example, invasive activity, proliferation) to HA presence are contradictory and can be specific to each immortal or patient-derived cell line [14,15].

The current research aims to study the biological consequences of HA exposure on 3D spheroids obtained from six unique transplantable tissue strains of high-grade brain tumors, including astrocytoma, oligodendroglioma, ependymoma, glioblastoma, and neuroma. The study assesses for the first time the effect of HA on neurosphere fusion rates and transcriptomic changes.

## 2. Materials and Methods

### 2.1. Cell Cultures

Cell cultures were obtained from transplanted tissue strains of laboratory animals’ brain tumors (Table 1) from the collection of Avtsyn Research Institute of Human Morphology of the Federal State Budgetary Scientific Institution “Petrovsky National Research Center of Surgery”, Moscow, Russia. This collection has the status of “Unique Scientific Installation”, assigned to objects of scientific infrastructure that allow obtaining significant scientific results and have no close analogues. Previously, the type identity of the tumors was confirmed by neuromorphologists using histological and immunohistochemical analysis, and the species identity was determined through PCR targeting mitochondrial genes and karyotype analysis.

Brain tumor tissue was washed with Hanks’ solution, minced, and dissociated through pipetting; the suspension was passed through a 70 μm pore size PES filter and transferred to a culture dish. DMEM/F-12 with 10% fetal calf serum and 1% penicillin-streptomycin (PanEco) was used as a culture media. The above experiment used cells of the second passage.

### 2.2. Spheroids

Cell cultures obtained from brain tumor tissue strains were detached from the substrate with Accutase^®^ enzymes (Himedia, Mumbai, India), the number of cells was estimated using the TC20 automated cell counter (Bio-Rad, Hercules, CA, USA), and then the culture was transferred into ultra-low attachment 60 mm Petri dishes (Corning, Corning, NJ, USA) at the rate of 10^5^ live cells per 1 mL of culture media (total 5 × 10^5^ cells per one dish), either supplemented with HA (SPL Life Sciences, FL, USA; to 2.5 mg/mL) or not. The cells were maintained for 2–4 days without medium change or agitation. Standard culture conditions of 37 °C, 5% CO_2_, and 95% humidity were maintained. Spheroids were formed through the self-aggregation of cells. Spheroids were live-imaged on culture day 2–4 using an Axiovert 40 CFL inverted microscope (Zeiss, Oberkochen, Germany) with AxioVs40 4.8.2.0 software (Zeiss). To estimate the sizes of individual spheroids or conglomerates of fused spheroids, their area was assessed in micrographs, taking at least 100 measurements for each 3D culture. For gene expression assay, spheroids were collected without the use of centrifugation (within 3–5 min, gravity forced the spheroids to sink to the bottom of the 15 mL centrifuge tubes), transferred to RNAlater (QIAGEN), incubated overnight at 4 °C, and stored at −80 °C until the analysis.

### 2.3. Spheroid Viability Assays

Spheroids obtained as mentioned above were cultured in 0.25% HA-supplemented or standard medium for 4 days. Then, spheroids were stained with mixed dye solution as follows: calcein AM (1 µM), Hoechst 33258 (5 µg/mL) and propidium iodide (2 µg/mL) for 30 min at 37 °C. Spheroids were live-imaged using a Leica DM 4000 B fluorescent microscope and LAS AF v.3.1.0 build 8587 software (Leica Microsystems, Wezlar, Germany).

Cell cultures obtained from brain tumor tissue strains were detached from the substrate with Accutase^®^ enzymes (Himedia, Mumbai, India), the number of cells was estimated using the TC20 automated cell counter (Bio-Rad, Hercules, CA, USA), and then the cell culture was transferred into 12-well ultra-low attachment plates (Corning) at the rate of 10^5^ live cells per 1 mL of culture media (total 2 × 10^5^ cells per one well), either supplemented with HA (SPL Life Sciences; to 2.5 mg/mL) or not. Growth medium without phenol red was used for this experiment. The cells were maintained for 4 h (day 0), 48 h (day 2), or 96 h (day 4) without medium change or agitation. Finally, 100 μL of the CCK-8 reagent (Servicebio, Wuhan, China) was added into each well, and the optical density (OD) at 450 nm was measured using a microplate reader (FlexA-200, Allsheng, Hangzhou, China) after incubation for 3 h at 37 °C. The results obtained were normalized to the OD of the standard or 0.25% HA-supplemented media without cells.

### 2.4. Gene Expression Analysis

Transcription rates of *Hif1a*, *Pdgfra*, *Tp53*, *Melk*, *Ecad*, *Ncad*, *Gdnf*, *Cd44*, *Cd133*, *Cdkn2a*, *Mki67*, *Pik3cg*, *Pten*, *Abcb1b*, and *Sox2* were assessed using real-time quantitative reverse transcription PCR. Four biological replicates were performed for each studied spheroid culture condition. The total RNA was isolated from RNA-later frozen samples using the RNeasy Plus Mini Kit (QIAGEN, Hilden, Germany). The estimated purified RNA concentration in the eluate was 0.1 g/L; good RNA integrity was confirmed using electrophoresis on 1% agarose gel stained with ethidium bromide. The synthesis of cDNA from the total RNA matrix was performed using a ready-made MMLV RT kit (Eurogen, Moscow, Russia). The obtained cDNA was PCRed in triplicate using the qPCRmix-HS SYBR reagent kit containing the fluorescent intercalating dye SYBR Green I (Eurogen). PCR primers were designed using the Primer-BLAST online resource in accordance with generally accepted requirements. The primers (Table 2) were synthesized by Eurogen.

Gene expression levels were quantified using the threshold cycle (Ct) method. Characteristic values were automatically generated using nonlinear regression analysis, and the relative expression values were calculated using an approach originally introduced by Pfaffl [16] using Gapdh as reference target, as it had exhibited the high expression stability under all tested conditions.

### 2.5. Statistical Analysis

The obtained data were analyzed using GraphPad Prism v.8.4.3. (GraphPad Software LLC) and StatTech v. 2.8.8 (StatTech, Saint Petersburg, Russia) software. Quantitative indicators were assessed for compliance with normal distribution using the Kolmogorov–Smirnov test. Two-group comparisons for normally distributed quantitative indicators with equal variances involved Student’s t-test. Two-group comparisons for quantitative indicators with distributions other than normal involved the Mann–Whitney U test. Correlations between two quantitative indicators were analyzed using the Spearman rank correlation test and the Chaddock scale for interpretation.

## 3. Results

### 3.1. Cell Cultures Derived from Tissue Strains of Brain Tumors

**Figure 1:**
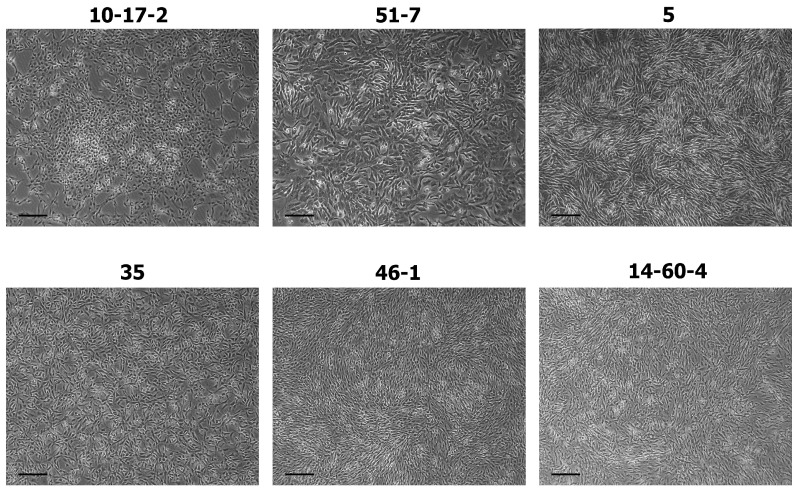
Cell cultures derived from tissue strains of carcinogen-induced high-grade brain tumors, passage 2, including 10-17-2 (rat astrocytoma), 51-7 (mouse oligodendroglioma), 5 (mouse ependymoma), 35 (rat glioblastoma), 46-1 (rat neuroma), and 14-60-4 (rat glioblastoma). Phase-contrast microscopy, scale bar 200 µm.

The tissue strain specimens were successfully converted into adhesive monolayer cultures of small spindle-shaped or polygonal cells. The patterns that the cells form in 2D culture were reminiscent of rosettes, mesh, or a wave-like structure. The cultures imaged in the second passage are shown in Figure 1.

### 3.2. Spheroids Derived from Cell Cultures

**Figure 2:**
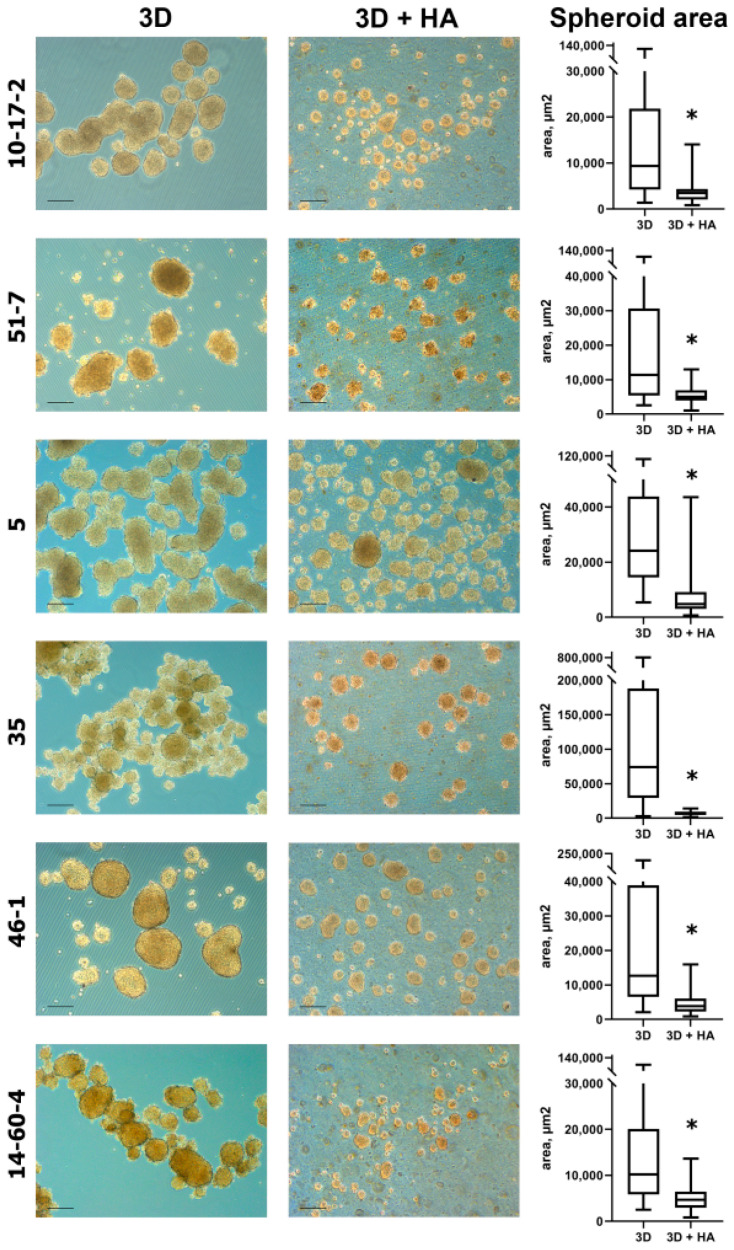
(on the left) Spheroids obtained from tumor cell cultures on day 2 (strain 35) or day 4 (other strains), cultured in 0.25% HA-supplemented vs. standard medium Phase-contrast microscopy, scale bar 200 µm. Spheroids as follows: 10-17-2 (rat astrocytoma), 51-7 (mouse oligodendroglioma), 5 (mouse ependymoma), 35 (rat glioblastoma), 46-1 (rat neuroma), and 14-60-4 (rat glioblastoma) (on the right). Spheroid area in micrographs taken from cultures grown in 0.25% HA-supplemented vs. standard medium. The data are presented as min, Q1, median, Q3, max, * *p* < 0.001.

The formation of spheroids began the very next day after transferring the cell suspension of the studied cultures into a culture dish with ultra-low adhesion. On culture day 2 (strain 35) or culture day 4 (other strains) in the standard medium, spheroids started to fuse into large conglomerates; however, when cultured in the HA-supplemented medium, the spheroids remained separate and uniform in size. HA did not completely exclude the fusion of neurospheroids because we still observed individual conglomerates, identified by their large size and irregular shape, but their number was single in the field of view. An assessment of the spheroids’ sizes in micrographs showed that the area of spheroids when cultured in HA-supplemented media was, in all cases, significantly smaller than when cultured in standard media, *p* < 0.001 (Figure 2).

### 3.3. Spheroid Viability Assays

**Figure 3:**
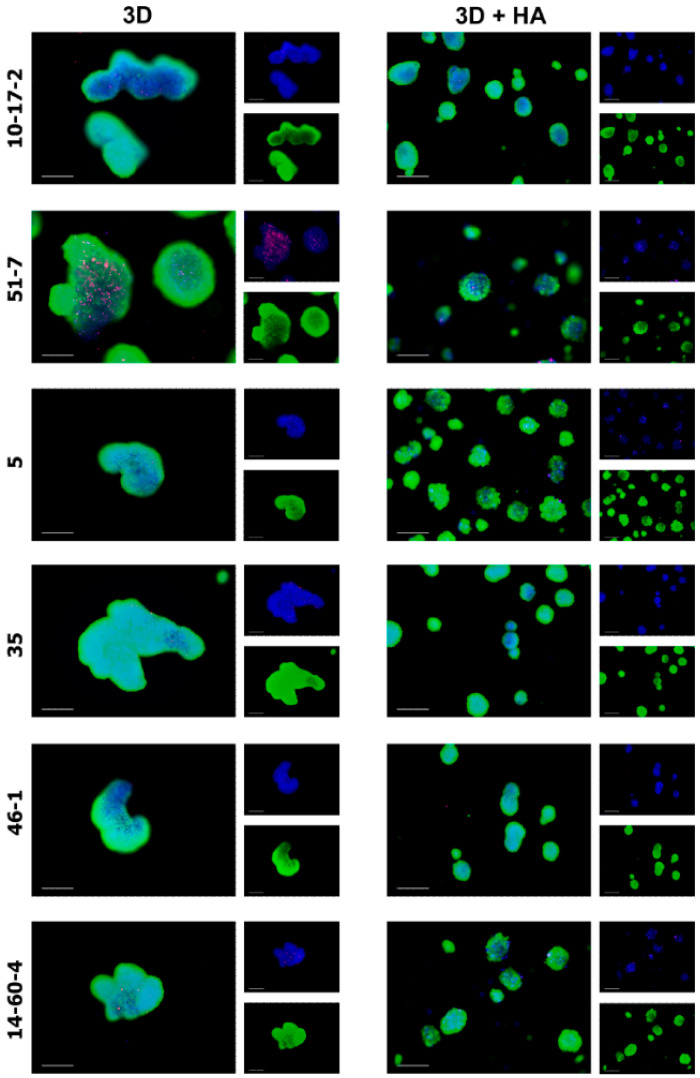
Spheroids obtained from tumor cell cultures on day 4, cultured in 0.25% HA-supplemented vs. standard medium. Spheroids are stained with mixed dye solution as follows: calcein AM (metabolically active cells, green), Hoechst 33258 (all cells, blue) and propidium iodide (dead cells, red). Fluorescent microscopy, scale bar 200 µm. Spheroids: 10-17-2 (rat astrocytoma), 51-7 (mouse oligodendroglioma), 5 (mouse ependymoma), 35 (rat glioblastoma), 46-1 (rat neuroma), and 14-60-4 (rat glioblastoma).

The vast majority of cells within the spheroids remained alive for 4 days and maintained the metabolic activity, converting calcein AM into green, fluorescent calcein. PI-positive cells were also present within the spheroids, but a quantification of the proportion of dead cells was difficult due to the high variability between individual spheroids (Figure 3).

**Figure 4:**
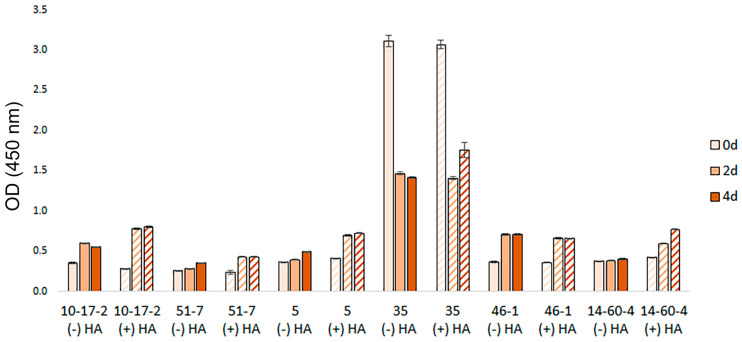
Spheroid viability measured using a CCK-8 assay on days 0, 2, and 4; spheroids were cultured in 0.25% HA-supplemented vs. standard medium. Spheroids: 10-17-2 (rat astrocytoma), 51-7 (mouse oligodendroglioma), 5 (mouse ependymoma), 35 (rat glioblastoma), 46-1 (rat neuroma), and 14-60-4 (rat glioblastoma). Data are presented as mean ± standard deviation.

A CCK8 assay confirmed that the cells maintained or increased their metabolic activity when 3D cultured for 4 days. There was one exception, which was that spheroids obtained from 35 glioblastoma cells had abnormally high activity of mitochondrial enzymes when only cell suspension was present in the medium (day 0, prior to spheroid formation). On days 2–4, 35 spheroids had decreased levels of metabolic activity, which nevertheless remained significantly higher than those of spheroids obtained from the other five brain tumors (Figure 4).

### 3.4. Gene Expression Profiling

**Figure 5:**
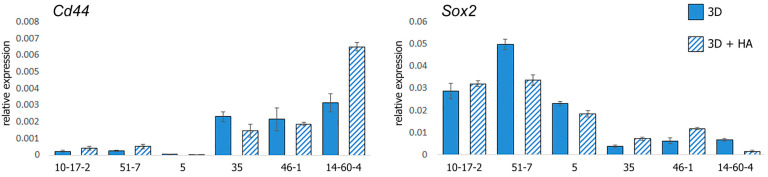
Relative expression of the Cd44 and Sox2 genes in spheroids. Spheroids: 10-17-2 (rat astrocytoma), 51-7 (mouse oligodendroglioma), 5 (mouse ependymoma), 35 (rat glioblastoma), 46-1 (rat neuroma), and 14-60-4 (rat glioblastoma). Data are presented as mean ± standard deviation.

The cultures were assigned into groups based on expression level of the hyaluronic acid receptor *Cd44*, including a *Cd44lo* group encompassing spheroid cultures 10-17-2, 51-7, and 5 with an order of magnitude-lower expression of *Cd44* than in the other group (*Cd44hi* spheroids 35, 46-1, and 14-60-4), *p* < 0.05 (Figure 5). We found that the two groups also differed by expression levels of *Sox2* transcription factor (Figure 5). The correlation analysis revealed a tight negative association (Chaddock scale) for *Cd44* and *Sox2* (ρ = −0.748, *p* < 0.01).

**Figure 6:**
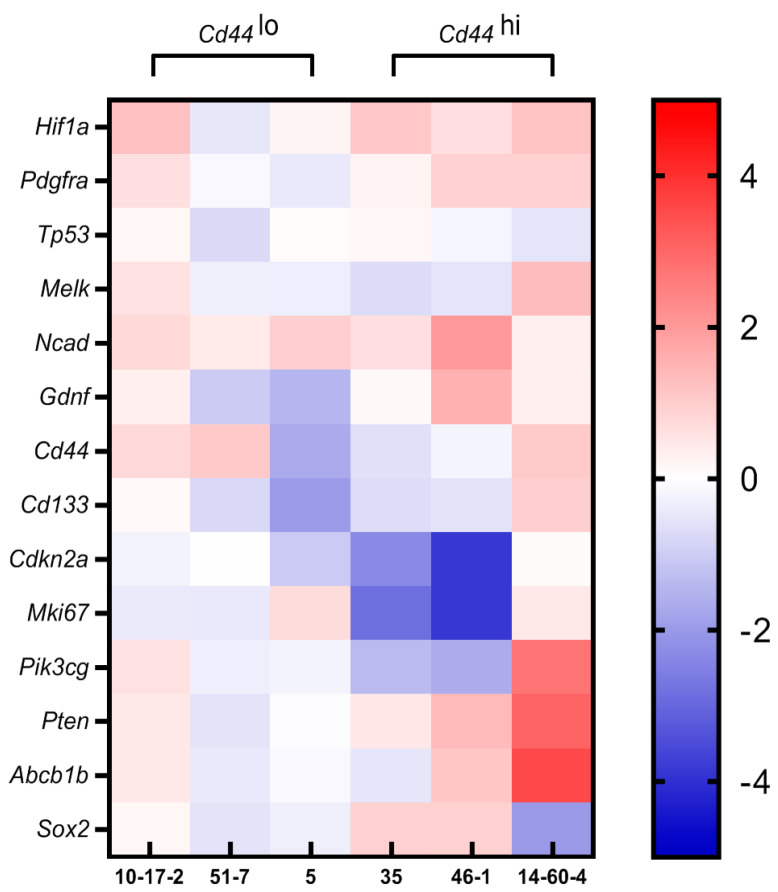
Heat map representing the log2 fold-change values for the 14 genes differentially expressed in spheroids cultured in 0.25% HA-supplemented vs. standard medium. Spheroids: 10-17-2 (rat astrocytoma), 51-7 (mouse oligodendroglioma), 5 (mouse ependymoma), 35 (rat glioblastoma), 46-1 (rat neuroma), and 14-60-4 (rat glioblastoma).

Next, we compared expression levels of particular genes in spheroids exposed to HA vs. spheroids cultured in the standard medium. A heat map representing log2 fold-change values for the 14 genes is shown in Figure 6. The *Cd44hi* group showed more specific and pronounced HA exposure-related transcriptomic changes compared with the other group, *Cd44lo.*

In *Cd44lo* spheroids derived from rat astrocytoma 10-17-2 and mouse oligodendroglioma 51-7, expression levels among 14 genes in response to HA changed by no more than threefold. In *Cd44lo* spheroids derived from ependymoma 5, most of the genes showed negligible reaction to HA, except *Cd44* and *Cd133*, with expression levels decreasing 3.3- and 4.0-fold, respectively, *p* < 0.05.

In *Cd44hi* spheroids, <3-fold changes were observed for seven genes, including *Hif1a*, *Pdgfra*, *Gdnf*, *Tp53*, *Melk*, *Cd44,* and *Cd133*. In spheroids derived from mouse gliobastoma 35 and rat neuroma 46-1, the expression levels of the *Cdkn2a* and *Mki67* genes were reduced manifold (glioblastoma 35 demonstrated a fivefold decrease in *Cdkn2a* and sevenfold decrease in *Mki67* expression, while neuroma 46-1 showed a fourteenfold decrease in both genes’ expression, *p* < 0.01); in addition, spheroids 46-1 demonstrated the decrease in *Pik3cg* expression and a more than fourfold increase in *Ncad* expression, *p* < 0.05. In Cd44hi spheroids derived from rat gliobastoma 14-60-4, HA-related transcriptomic patterns differed from all other strains. *Ncad*, *Hif1a*, *Pdgfra*, *Gdnf*, *Tp53*, *Melk*, *Cd44*, *Cd133*, *Cdkn2a* and *Mki67* showed <3-fold changes; at that, *Sox2* expression decreased 4.0-fold, *p* < 0.05, while *Pik3cg*, *Pten*, and *Abcb1b* expression increased 6.6-, 8.1-, and 11.6-fold, respectively, *p* < 0.01.

## 4. Discussion

The most pronounced effect that we observed when adding HA to the culture media was a significant inhibition of the spheroid fusion. The ability of neurospheres to spontaneously aggregate during culture is a long-known phenomenon [17]. The capabilities of individual cell elements to aggregate into larger structures are much exploited in 3D bioprinting of normal and tumor tissues [18]. The method affords 3D shapes through high precision layering of bioink—biocompatible hydrogels loaded with single cells or cell spheroids. Of note, a very important part of the process is ensuring the generation of spheroids of uniform size and shape, as well as the ability to control the fusion between adjacent spheroids into prescribed and stable structures during culture [19,20]. This can be partially ensured by the hydrogel itself, which modulates the ability of spheroids to aggregate, presumably by changing the difference in interfacial surface tension between the surrounding culture media and cells [20]. Among many hydrogel precursor materials, HA stands out due to its biocompatibility, hydrophilicity, non-immunogenicity, and complete biodegradability [20,21]. At that, relatively few studies analyze the influence of HA on spheroid fusion rates. For example, an experiment using an in vitro model of knee cartilage showed that chondrospheres retained their fusion capability when exposed to high-viscosity hylan-based hydrogel (hylan is a chemically crosslinked form of HA) [22]. Another study revealed that 0.4% HA of all tested molecular weights within the range of 60–1000 kDa ensured a uniform size distribution of spheroids derived from primary human dermal fibroblasts [23]. It is known that the efficiency of spheroid fusion, in addition to culture conditions, also depends on the type of cells themselves, the rates of ECM production, and the formation of a condensation boundary with time [24]. The property of HA to prevent the fusion of brain-derived spheroids identified by us in this study can be employed in CNS regenerative medicine and experimental oncology to ensure the production of uniform, controllably fusing neurospheres when creating in vitro brain tumor models. Currently, for bioprinting brain models using the extrusion method, combinations of tumor and tumor-associated cells (endothelial or immune) and bioink in the form of hydrogels based on gelatin, sodium alginate, collagen type 1, fibrinogen, and decellularized extracellular matrix are used [25]. In our opinion, HA-containing hydrogels provide a more physiologically relevant microenvironment, which renders it promising for use in personalized medicine to examine the efficacy of therapeutic agents.

We performed viability tests to confirm that the decrease in spheroid diameter and fusion rate was not caused by cell death in response to the presence of HA. Both PI-calcein AM staining and the CCK8 assay showed that the cells retain their metabolic activity within spheroids when cultured in both standard and HA-supplemented media. Moreover, the CCK8 test revealed abnormally high mitochondrial enzyme activity in cells derived from 35 glioblastoma. This observation can be explained by the fact that mutations in genes encoding NADP+-dependent isocitrate dehydrogenases (especially IDH1) are common in lower-grade diffuse gliomas and secondary glioblastomas, thus providing increased mitochondrial activity of cancer cells [26].

We hypothesized that a decrease in the intensity of spheroid fusion in the presence of HA may be associated with a change in the expression level of cadherins, which can play an important role in this process [27]. A PCR assay showed an almost complete absence of E-cadherin in all studied samples, while N-cadherin was highly expressed by tumor cells. This observation is consistent with published data that in brain tumors the expression of E-cadherin is suppressed, while the level of N-cadherin is up-regulated; immunohistochemistry performed in 60 human ependymomas revealed about 77% of N-cadherin-positive and only 2% of E-cadherin-positive cases [28]. Another study examining brain tumors found similar results; the expression of E-cadherin was observed in 8/92 gliomas with weak staining intensity in the majority of the immunoreactive cases (7/8), while the expression of N-cadherin was identified in 81/92 cases, although the correlation between presence of both cadherin expression and WHO tumor grades (low-grade vs. high-grade) was not significant statistically [29]. Much to our surprise, the N-cadherin level increased even more when spheroids were cultured in an HA-supplemented medium; this observation requires further study because it suggests other mechanisms for controlling the fusion of individual spheroids with each other.

HA is the main ligand of CD44 transmembrane glycoprotein; its binding triggers the release of the intracellular domain of CD44 and its translocation into the nucleus, where it acts as a transcription factor, activating the expression of genes involved in cell adhesion, proliferation, invasion, and metastasis [14,30]. A high level of *CD44* in a tumor correlates with a poor survival prognosis for patients with various cancers, including brain tumors, so it is often considered as a marker of tumor stem cells [30,31,32], although this assumption is questioned by some researchers [33]. Notably, the protein expression level of CD44 in grade II and III gliomas is higher than that in normal brain tissue but lower than that in glioblastoma grade IV tissue [32]. Corresponding to these data, the CD44 cell surface receptor is expressed by many immortalized human and rodent glioma cell lines [30,31,33], but its levels may vary dramatically, even between single cell-derived colonies of the same line, e.g., up to 20-fold for U-251 MG glioblastoma [34]. The observed difference in *Cd44* expression among spheroids derived from different tumor strains is consistent with the notorious clinical and molecular heterogeneity of gliomas; the expression of *CD44* is associated with age, chemotherapy, grade, and primary/recurrent/secondary tumor type of gliomas in humans [32].

*Sox2* expression in brain tumor tissues is characteristic of both stem and differentiated cells; it is necessary to maintain a sufficient level of plasticity, allowing their transition between these states [35]. Interestingly, significantly higher percentages of *SOX2*⁺ cells were indicated at the infiltrating brain tumor edge when compared with other areas (necrotic tumor, viable solid tumor, peritumoral normal brain, normal brain close to the tumor and normal brain distant from the tumor), thus confirming that the edge of the tumor is the moving front for tumor progression and invasion [35]. Experimentally, we revealed a tight negative association (Chaddock scale) between *Cd44* and *Sox2* gene expression levels. In most studies, researchers, on the contrary, note a positive correlation between the expression of the *CD44* and *SOX2* genes; thus, the U251-MG glioma clone with the highest *CD44* expression also had the highest *SOX2* level among the five obtained clones in the experiment mentioned above [34]. On the other hand, and consistently with our data, Abugomaa et al. report oppositely directed expression dynamics for *Cd44* and *Sox2* during a 3- to 2D transition in canine urothelial carcinoma-derived organoids, a 20- to 35-fold increase for *Cd44* (depending on the strain) and a 20-fold decrease for *Sox2* [36]. Interestingly, both *CD44* and *Sox2* are markers of radiation resistance in primary glioblastoma cultures, but *CD44* expression correlates positively with radioresistance, while the *Sox2*-positive subpopulation shows the highest radiosensitivity [37]. In our opinion, the identified negative association between *Cd44* and *Sox2* will require further investigation because it indirectly indicates the presence of several types of cells with stem characteristics in brain tumors.

For further analysis, the cultures were distributed into two groups based on *Cd44* expression. We assumed that *Cd44lo* and *Cd44hi* spheroids might differ in their response to HA. Accordingly, we comparatively assessed the HA-induced transcriptomic changes in *Cd44lo* and *Cd44hi* spheroids for a panel of genes involved in the occurrence and progression of brain tumors.

The *Cd44lo* spheroids showed a weak transcriptomic response to HA exposure, with subtle changes for all 14 genes in spheroids derived from astrocytoma 10-17-2 and oligodendroglioma 51-7 and a 34-fold decrease for Cd44 and Cd133 only in spheroids derived from ependymoma 5. Of note, the ependymoma 5 spheroids had the lowest baseline expression levels of *Cd44* and *Cd133* in the entire sample of six tumor strains.

The *Cd44hi* spheroids showed a more pronounced transcriptomic response to HA compared with the other group. Moreover, in spheroids obtained from glioblastoma 35 and neuroma 46-1, these changes were predominantly unidirectional, and the most pronounced decrease was shown by the *Cdkn2a* and *Mki67* genes involved in the regulation of proliferation and the cell cycle. It is important to note that most studies demonstrate that HA stimulates the proliferation of both normal and tumor cells in vitro; however, some studies show the opposite effect, which is that under certain conditions, HA is able to suppress the proliferative activity of amniotic epithelial cells, vascular smooth muscle cells, fibroblasts, breast cancer cells, osteosarcoma cells, and colon cancer cells [38]. The authors suggest that such cell-specific effects may originate from the special properties of the cells, perhaps by the initial ratio of enzymes involved in HA synthesis and degradation, the level of expression of HA receptors, or other characteristics of the cell culture or line [39].

By contrast, in spheroids derived from glioblastoma 14-60-4, the *Cdkn2a* and *Mki67* mRNA levels were unchanged, and the transcription rates for *Pik3cg*, *Pten*, and *Abcb1b* increased dramatically, while *Sox2* expression decreased, reaching minimal values recorded in the whole experiment. These dynamics may indicate decreased plasticity of tumor cells in 14-60-4 spheroids, accompanied by simultaneous activation of the PI3K/AKT/mTOR pathway, a major survival pathway activated in cancer. To date, efforts to develop targeted therapies have been unsuccessful for reasons including extensive internal intrapathway or external interpathway negative feedback loops [40]. The *PTEN* (phosphatase and tensin homolog) is a tumor suppressor and the major brake of the pathway. This pattern is consistent with recent evidence that activated PI3K signaling, both physiological and oncogenic, upregulates the expression of its negative regulator *PTEN*, thereby limiting the signal duration and the regulatory output of the pathway [41].

Even more relevant, in our opinion, is the 12-fold increase in transcription levels for *Abcb1b* (also known as multidrug resistance 1, *Mdr1*) encoding the ABCB1 drug efflux transporter of the blood–brain barrier, which limits the delivery of drugs to the brain. The upregulation of the ABCB1 transporter and activation of the PI3K pro-survival and antiapoptotic pathway occurred simultaneously in 14-60-4 spheroids. Similar results were obtained in the work of Misra et al., who showed that the constitutive interaction of the hyaluronan with its CD44 receptor in MCF-7/Adr human breast carcinoma cells activates the PI3K pathway, enhancing the expression of ABCB1/MDR1 and thus contributing to treatment resistance [42]. It is also known that HA-CD44 interactions activate a variety of signaling pathways (including the PI3K pathway) that contribute to stimulation of ABCB1 expression, leading to drug resistance in breast and ovarian tumor cells [43] and bone tumor cells [44]. An experiment with the immortalized glioblastoma U-118 MG line revealed that the interaction of cells with the 3D HA-chitosan scaffold made them more resistant to chemotherapy, which corresponded to the increased expression of drug efflux transporter [45]. Strategies for overcoming ABCB1 action have been largely unsuccessful, which poses a tremendous clinical problem to treat central nervous system diseases [46]. In our settings, only one of the six cultures showed a similar response to HA; however, this observation may be elaborated in subsequent research taking into account the recently developed concept that considers HA as a potential target in anticancer therapy [47]. Moreover, HA-based materials are attractive systems for the effective delivery of antitumor agents. Numerous studies have demonstrated the ability of HA to target *CD44*-overexpressing cancer cells [48]; however, it must be remembered that the level of *CD44* expression can differ by an order of magnitude depending on the tumor, as we have showed in our study.

It is noteworthy that none of the studied tumor spheroids reacted to HA by the altered expression of the most crucial markers in brain tumor pathogenesis, for example, *Hif1a* and *Pdgfra,* which control tumor growth in normoxia or mild hypoxia [49], the tumor suppressor *Tp53* [50], or the oncogenes *Melk* and *Gdnf* [51]. It can be assumed that HA content (in media for cell cultures or presumably, in ECM for brain tumors) has low involvement in these tumor-associated regulatory networks.

To sum up, the results of our study show that 3D spheroids obtained from high-grade carcinogen-induced brain tumors are prone to spontaneous fusion, the rates of which can be significantly reduced by HA. When exposed to 0.25% HA in culture medium, spheroids effectively retained their size and shape uniformity. During the HA exposure, spheroids with low levels of HA receptor Cd44 underwent subtle transcriptomic changes, whereas Cd44hi spheroids reacted by the multifold activation/repression of genes involved in the regulation of the cell cycle, PI3K/AKT/mTOR signaling, and multidrug resistance. The potential HA-induced increase in brain tumor 3D models’ resistance to anticancer drug therapy should be taken into account when designing preclinical studies using HA scaffold-based models. The property of HA to prevent the fusion of brain-derived spheroids can be employed in CNS regenerative medicine and experimental oncology to ensure the production of uniform, controllably fusing neurospheres when creating more accurate in vitro brain models.

## Figures and Tables

**Table 1:** Transplantable tissue strains of high-grade brain tumors.

Tissue Strain	Tumor Type	IHC Markers	Animal Species	Carcinogen
10-17-2	Astrocytoma	GFAP+ ATRX− CD68−	Rattus norvegicus, rat	N-ethyl-N-nitrosourea
51-7	oligodendroglioma	ATRX+	Mus musculus, mouse	7,12-Dimethylbenzathracene
5	Ependymoma	GFAP+ CD99+	Mus musculus, mouse	7,12-Dimethylbenzathracene
35	Glioblastoma	Iba1+ CD68+ Cathepsin B+	Rattus norvegicus, rat	N-Nitrosomethylurea
46-1	Neuroma	Calretinin+ CD56+ S-100+	Rattus norvegicus, rat	N-ethyl-N-nitrosourea
14-60-4	Glioblastoma	Iba1+ CD68+	Rattus norvegicus, rat	N-ethyl-N-nitrosourea

**Table 2:** Oligonucleotide primer sequences.

Gene	Rat	Mouse
*Hif1a*	(5′ to 3′) TCACAGTCGGACAACCTCAC (3′ to 5′) TGCTGCAGTAACGTTCCAATTC	(5′ to 3′) AGGATGAGTTCTGAACGTCGAAA (3′ to 5′) CTGTCTAGACCACCGGCATC
*Pdgfra*	(5′ to 3′) ATTGTGCTCCGTAGTCCCCA (3′ to 5′) TCCTAGCCAGGGATCTTGCC	(5′ to 3′) GTGCTAGCGCGGAACCT (3′ to 5′) CATAGCTCCTGAGACCCGC
*Tp53*	(5′ to 3′) AGGGAGTGCAAAGAGAGCAC (3′ to 5′) GTCTTCGGGTAGCTGGAG G	(5′ to 3′) TTCTCCGAAGACTGGATGACTG (3′ to 5′) TCCATGCAGTGAGGTGATGG
*Melk*	(5′ to 3′) TGCCTTGGGCAGAAAAACAGAT (3′ to 5′) GGCCTGCAGGTCGGATACT	(5′ to 3′) ACCAAGAAAGCGAAAGCTGC (3′ to 5′) CGAAGTCCACGTTCTTCTTTGG
*Ncad*	(5′ to 3′) ACCCAGGAAAAGTGGCAGGT (3′ to 5′) TCGGAGGGATGACCCAGTCT	(5′ to 3′) TGTGGAGGCTTCTGGTGAAAT (3′ to 5′) TCTCACAGCATACACCGTGC
*Ecad*	(5′ to 3′) CCTTCCTCCCAATACATCTCCC (3′ to 5′) TCTCCGCCTCCTTCTTCATC	(5′ to 3′) GGTTTTCTACAGCATCACCG (3′ to 5′) GCTTCCCCATTTGATGACAC
*Gdnf*	(5′ to 3′) TATGGGATGTCGTGGCTGTCT (3′ to 5′) AGTCACTGGTCAGCGCGAAG	(5′ to 3′) GACCGGATCCGAGGTGC (3′ to 5′) GAGGGAGTGGTCTTCAGCG
*Cd44*	(5′ to 3′) TTTATTGGGAGCACCCTGGC (3′ to 5′) AGGGGTAGTCATCAAGGCTGT	(5′ to 3′) AGAAGGGACAACTGCTTCGG (3′ to 5′) TTGGAGCTGCAGTAGGCTG
*Cd133*	(5′ to 3′) AATTCAAAGCCCCGGGAGTG (3′ to 5′) CTGGGGGATCAGGCCAACTA	(5′ to 3′) GGAGCAGTACACCAACACCA (3′ to 5′) GTCTGTTTGATGGCTGTCGC
*Cdkn2a*	(5′ to 3′) GTACCCCGATACAGGTGATGAT (3′ to 5′) GATACCGCAAATACCGCACG	(5′ to 3′) TGGTCACTGTGAGGATTCAGC (3′ to 5′) TGCCCATCATCATCACCTGG
*Mki67*	(5′ to 3′) TTCCAGACACCAGACCATGC (3′ to 5′) GGGTTCTAACTGGTCTTC TGG	(5′ to 3′) CCTGCCTGTTTGGAAGGAGT (3′ to 5′) AAGGAGCGGTCAATGATGGTT
*Pik3cg*	(5′ to 3′) TGGATATGAAGGGAGCCCCA (3′ to 5′) CATGCCCTAGGTGACCTGAC	(5′ to 3′) CCGCTCAGGGAGAGGAGTA (3′ to 5′) CCACTCTCAGCTTCACCTCC
*Pten*	(5′ to 3′) GCCAAATTTAACTGCAGAGTTGC (3′ to 5′) GCGCCTCTGACTGGGAATAG	(5′ to 3′) GGACCAGAGACAAAAAGGGAGT (3′ to 5′) CCTTTAGCTGGCAGACCACA
*Abcb1b*	(5′ to 3′) GTCCACCATCCA AACGCAG (3′ to 5′) GCACCTCAAATACTCCCAGCT C	(5′ to 3′) CTCTTGAAGCCGTAAGAGGCT (3′ to 5′) AACTCCATCACCACCTCACG
*Sox2*	(5′ to 3′) TCCATGGGCTCTGTG GTCAA (3′ to 5′) CATGTGCAGTCTACTGGGCG	(5′ to 3′) AGGAAAGGGTTCTTGCTGGG (3′ to 5′) GGTCTTGCCAGTACTTGCTCT
*Gapdh*	(5′ to 3′) CAGGGCTGCCTTCTCTTGTG (3′ to 5′) GCCTTGACTGTGCCGTTGAA	(5′ to 3′) AACTCAGGAGAGTGTTTCCTCG (3′ to 5′) TTTGCCGTGAGTGGAGTCAT

## Data Availability

Data is contained within the article.
